# Obituary

**Published:** 1874-11

**Authors:** A. K. Van Horn

**Affiliations:** Secretary; Freeport, Ill.


### Obituary.

FREEPORT, Ill., Sept. 28th, 1874.

At a special meeting of the Stephenson County Medical Society, convened at the office of Dr. Buckley, September 28th, 1874, to take action on the death of Dr. S. R. BUCHER, the following resolutions were adopted :

WHEREAS, we have been called to mourn the death of Dr. S. R. BUCHER, of Cedarville, a member of this Society ;

*Resolved*, That in his death this Society has lost a most efficient member, and one intimately identified with its best interests ; that the profession at large has lost one of its brightest ornaments, and our community a most useful citizen.

*Resolved*, That we believe his death was the result of too arduous labors in his chosen calling, and that he has added another to the long list of glorious martyrs who have given up their lives in the cause of suffering humanity.

*Resolved*, That while his family must most deeply mourn his loss, they have the consolation of knowing that the multitudes of sick and afflicted, whose sufferings his skillful hands have alleviated, and whose drooping hopes his encouraging words have cheered, will not soon, nor willingly let his name be forgotten.

*Resolved*, That we, as a Society, tender to the bereaved wife and orphaned family our heartfelt sympathy in this their hour of trial, trusting that the "Lord will temper the wind to the shorn lamb."

*Resolved*, That a copy of these resolutions be tendered to the family, and sent for publication to the journals of the city, and CHICAGO MEDICAL JOURNAL.

A. K. VAN HORN, *Secretary.*

